# Use of sulphur hexafluoride microbubbles injection to identify the sentinel lymph node in breast cancer patients: initial experience in a UK breast unit

**DOI:** 10.1186/bcr3766

**Published:** 2015-11-05

**Authors:** Alice Leaver, Simon Lowes, Peter Newton, Linsley Lunt, Anuradha Anand, Linda Hutchinson, Sally Athey, Amanda Jane Potterton, Sheetal Sharma, Alan Redman

**Affiliations:** 1 Gateshead Hospitals NHS Trust, Gateshead, UK

## Introduction

In our Trust, all breast cancer patients undergo preoperative axillary staging with ultrasound. Over the past year we have introduced intradermal sulphur hexafluoride microbubbles ultrasound contrast injection to help identify sentinel lymph nodes for a preoperative needle biopsy in each patient. Only patients with malignant node morphology on grey-scale ultrasound undergo biopsy without microbubbles injection.

## Methods

Prospective audit of data collated at the time of the microbubbles procedure together with multidisciplinary meeting records identified relevant screening and symptomatic patients with primary breast cancer treatment including axillary node surgery between 1 July 2014 and 1 July 2015. Descriptive statistics were performed.

## Results

Sixty-four female patients underwent microbubbles injection and axillary node surgery. Overall combined sensitivity and specificity of microbubbles ultrasound/biopsy procedure were 67% (8/12) and 100% (52/52) respectively. Seventy-five per cent of operative sentinel node biopsies (45/60) showed evidence of previous needle biopsy (four axillary clearance specimens excluded). Needle biopsy detection of micrometastatic disease only, shortly after commencing microbubbles use, led to multidisciplinary meeting consideration of size of needle biopsy metastasis and ultrasound appearance of sentinel and surrounding nodes in triage of patients to type of axillary surgery. Results represent the combined learning curve of seven radiologists. The procedure was well tolerated by patients and technically easy to perform. The greatest challenges were optimising ultrasound machines for microbubbles visualisation, and finding time within busy clinics to perform the procedure.

## Conclusion

In this small patient cohort, introduction of microbubbles has facilitated reliable and effective identification of the sentinel lymph node for assessment of morphology on ultrasound and also biopsy.

